# A group LASSO-based method for robustly inferring gene regulatory networks from multiple time-course datasets

**DOI:** 10.1186/1752-0509-8-S3-S1

**Published:** 2014-10-22

**Authors:** Li-Zhi Liu, Fang-Xiang Wu, Wen-Jun Zhang

**Affiliations:** 1 Department of Mechanical Engineering, University of Saskatchewan, 57 Campus Drive, S7N 5A9 Saskatoon, Canada; 2 Division of Biomedical Engineering, University of Saskatchewan, 57 Campus Drive, S7N 5A9 Saskatoon, Canada

**Keywords:** Gene Regulatory Network, Reverse Engineering, Group LASSO, Optimization, Gene Expression Data

## Abstract

**Background:**

As an abstract mapping of the gene regulations in the cell, gene regulatory network is important to both biological research study and practical applications. The reverse engineering of gene regulatory networks from microarray gene expression data is a challenging research problem in systems biology. With the development of biological technologies, multiple time-course gene expression datasets might be collected for a specific gene network under different circumstances. The inference of a gene regulatory network can be improved by integrating these multiple datasets. It is also known that gene expression data may be contaminated with large errors or outliers, which may affect the inference results.

**Results:**

A novel method, Huber group LASSO, is proposed to infer the same underlying network topology from multiple time-course gene expression datasets as well as to take the robustness to large error or outliers into account. To solve the optimization problem involved in the proposed method, an efficient algorithm which combines the ideas of auxiliary function minimization and block descent is developed. A stability selection method is adapted to our method to find a network topology consisting of edges with scores. The proposed method is applied to both simulation datasets and real experimental datasets. It shows that Huber group LASSO outperforms the group LASSO in terms of both areas under receiver operating characteristic curves and areas under the precision-recall curves.

**Conclusions:**

The convergence analysis of the algorithm theoretically shows that the sequence generated from the algorithm converges to the optimal solution of the problem. The simulation and real data examples demonstrate the effectiveness of the Huber group LASSO in integrating multiple time-course gene expression datasets and improving the resistance to large errors or outliers.

## Background

A Gene regulatory network (GRN) consists of a set of genes and regulatory relationships among them. Tremendous amount of microarray data that measure expression levels of genes under specific conditions are obtained from experiments. It is a challenging problem in systems biology to reconstruct or "reverse engineer" GRNs by aiming at retrieving the underlying interaction relationships between genes from microarray data. Various approaches have been developed to infer GRNs from microarray data. Most of them can be classified into two categories: parametric or model-based methods and nonparametric or dependency-measure-based methods. Commonly used models include ordinary differential equations [1], Gaussian graphical models [2] and Bayesian networks [3]. Dependency measures include partial correlation coefficient [4], mutual information [5], and *z*-score [6].

The reconstruction of GRN is a non-trivial problem. On the one hand, the number of possible network topologies grows exponentially as the number of genes increases. On the other hand, the information in the microarray data is quite limited. The data contain a lot of inherent noises generated from the devices or the experiment processes. For large-scale networks, the number of observations is usually much less than that of genes, also known as "dimensionality problem" [2,7]. The lack of observations and the high dimensionality of the data prohibit the direct application of traditional methods and make the inference task extremely challenging.

As more and more microarray datasets on the same species are produced from different laboratories, their integration leads to more robust and more reliable results. The methods that integrate multiple datasets could synergize the strength of each dataset and either infer a more accurate network if all the integrated datasets are in high qualities or infer a robust network which is better than the worse one that is from a single dataset. However, multiple time-course datasets can not be simply combined as one dataset as there is no temporal dependencies between the datasets. Wang et al. [8] proposes a linear programming framework to integrate multiple time-course gene expression data to infer a network topology that is most consistent to all datasets. In their method, the regulatory strengths between genes is assumed to be the same across all datasets. However, different datasets may be produced under different circumstances, which may result in different regulatory strength between genes. Another problem is that the value of the tuning parameter in their method, which controls the degree of sparsity of the inferred network, is only determined intuitively. Chen et al. [9] infer one GRN from each time-course data separately, and combine edges of inferred GRNs using a strategy similar to majority vote. For this method, using each dataset separately in the inference process may miss the opportunity of taking advantage of information in other datasets and the tuning parameter is also determined intuitively.

This study focuses on inferring the topologies of GRNs from multiple time-course gene expression datasets based on an autoregressive model. We assume that one GRN corresponds to one dataset and these GRNs share the same topology across all datasets. By assigning the parameters representing the regulatory strengths of the same edge into the one group, the group LASSO [10] can be applied to find the sparse network topology. Microarray data typically contain noises and outliers, which could severely affect the quality of inferred results. Rosset and Zhu [11] proposes a robust version of LASSO by replacing the squared error loss of LASSO with Huber loss. We propose to use the Huber loss to extend the group LASSO such that the new method, Huber group LASSO, is more resistant to the large noises and outliers.

To solve the Huber group LASSO, a new algorithm is developed in our previous work [12], which combines the idea of auxiliary function minimization [13] and the block coordinates descent method [14]. The proposed algorithm is efficient and can also be adapted for solving the group LASSO problem without the orthogonality restriction. In this study, we analyze the convergence of our proposed algorithm and show that the sequence the algorithm generated indeed converges to the optimal solution of the Huber group LASSO problem. Instead of picking a specific value for the tuning parameter which corresponds to a determinant network topology as in our previous work [12], in this study, we adapt the "stability selection" [15] strategy to our method to find a network consisting of edges with probabilities or scores. The Huber group LASSO is applied to both simulation data and real experimental data and its performances are compared with those of the group LASSO in terms of areas under the receiver operating characteristic (AUROC) and areas under the precision-recall (AUPR). Results show that the Huber group LASSO outperforms the group LASSO and therefore demonstrate the effectiveness of our proposed method.

Briefly, the remainder of the paper is organized as follows. In Model Section, we introduced the model for the GRN, based on which the network topology is inferred. In Result Section, our proposed method is applied to the both simulation data and real experimental data. The results demonstrate the effectiveness of our method. Then, we conclude this study and point out the future work along this research in Conclusion Section. Details of the method and its theoretical analysis can be found in Method Section.

## Model

A model for GRN consisting of *p *genes is used in this study [16]:

(1) $$\begin{array}{c} \dot{x}=\text{Cx}+\text{Sr},\\ \text{r}=\text{f}\left(\text{x}\right), \end{array}$$

where $x={\left[x_{1},\ldots ,x_{p}\right]}^{T}\in R^{p}$ is the vector of mRNA concentrations; $C=\text{diag}\left[-c_{1},\ldots ,-c_{p}\right]\in R^{p\times p}$ is a diagonal matrix with *c_i _>*0 the degradation rate of gene *i*; the vector $r={\left[r_{1},\ldots ,r_{m}\right]}^{T}\in R^{m}$ represents the reaction rates, which is a function of mRNA concentrations and $S\in R^{p\times m}$ is the stoichiometric matrix of the network. We assume that reaction rate **r **is a linear combination of mRNA concentrations,

(2) r=Fx,

where $F\in R^{m\times p}$. Then, (1) becomes

(3) $$\dot{x}=Cx+Mx,$$

where $M=SF\in R^{p\times p}$. The elements of **M **= (*m_ij_*)_1≤*i, j*≤*p *_indicate the network topology or regulatory relationships between genes. *m_ij _≠ *0 if gene *j *regulates the expression of gene *i*. Otherwise, *m_ij _*= 0, gene *j *does not regulate gene *i*.

Since the gene expression levels are sampled at several time points, by using zero order hold discretization method, system (3) is discretized as

(4) $$x_{k}=Ax_{k-1}$$

where **A **= *e*^**C**Δ*t *^+ **C**^−1^(*e*^**C**Δ*t *^*− ***I**)**M**. Note that *e*^**C**Δ*t*^and **C**^−1^(*e*^**C**Δ*t *^*− ***I**) are both diagonal matrices and their diagonal elements are all positive. Thus, the off-diagonal elements of **A **= (*a_ij_*)_1*≤i,j≤p *_have the same zero and nonzero pattern as those of **M**. In this study, we focus on inferring relationships between genes and do not consider self-regulations. As mentioned above, this can be achieved by identifying the nonzero off-diagonal elements in matrix **A**, which can be interpreted as regulatory strengths. Multiple time-course gene expression datasets for a GRN may be collected under different circumstances. One dataset is assumed to correspond to one inferred GRN topology, and all inferred GRNs should share the same network topology as their corresponding datasets are generated from the same underlying gene network. Our purpose is to reverse engineer the underlying network topology from these multiple datasets. More specifically, suppose we have *m *time-course gene expression datasets for a gene network: $\tilde{X}\left(1\right),\ldots ,\tilde{X}\left(m\right)$, each of which is measured at *n_k _*+1 time points, i.e., $\tilde{X}\left(k\right)\in R^{p\times \left(n_{k}+1\right)}$. According to the model (4), these datasets should satisfy

(5) Y(k)=A(k)X(k)+E(k),k=1,…,m,

where $Y\left(k\right)=\left[{\tilde{x}}_{2}\left(k\right),\ldots ,{\tilde{x}}_{n_{k}+1}\left(k\right)\right]$, the last *n_k _*observations; $X\left(k\right)=\left[{\tilde{x}}_{1}\left(k\right),\ldots ,{\tilde{x}}_{n_{k}}\left(k\right)\right]$, the first *n_k _*observations, $A\left(k\right)\in R^{p\times p}$, the regulatory matrix for the *k*th dataset and **E**(*k*), the errors or noises. All **A**(*k*)'s are required to have the same structure. i.e., zero and nonzero pattern, but do not need to have the same value for every nonzero position because gene network is dynamic and regulatory strength may be different under different circumstances. In this study, we propose to use group LASSO penalty to implement this requirement and to use Huber loss function to take into account the robustness. Details of the proposed method are shown in the Method Section.

## Results

To study the effectiveness of the proposed method, the Huber group LASSO is applied to inferring GRNs from both simulation datasets and real experimental datasets and the results of Huber group LASSO are compared with those from group LASSO in both area under receiver operating characteristic (AUROC) curve and area under the precision and recall (AUPR) curve.

### Simulation example

A small-GRN consisting of 5 genes is considered in this example. The corresponding true network topology matrix is

$$A_{0}=\left[\begin{array}{ccccc} + & - & + & 0 & 0\\ - & + & 0 & 0 & +\\ 0 & + & + & 0 & 0\\ + & - & 0 & + & 0\\ 0 & 0 & 0 & + & + \end{array}\right],$$

where + and *− *indicate the existence and regulation types of the edge. We randomly generate *m *stable regulatory matrices **A**(*k*), *k *= 1*, . . . , m*, according to the template **A**_0_, such that sign(**A**(*k*)) = sign(**A**_0_). Then, *m *simulated time-course gene expression datasets, each with the number of time points, *n_k _*, are generated from (5) with randomly chosen expression values at the first time point. The simulated error follows a mixed Gaussian distribution: with probability of 0.8, it has the distribution *N *(0, 1) and with probability of 0.2, it has the distribution *N *(0, 102). In this way, the simulated data contain large errors and outliers. To investigate the performances of our methods in different situations, we vary the values of *m *and *n_k _*and apply the group LASSO and Huber group LASSO respectively to these generated datasets and compare the results from these two methods.

**Figure 1:**
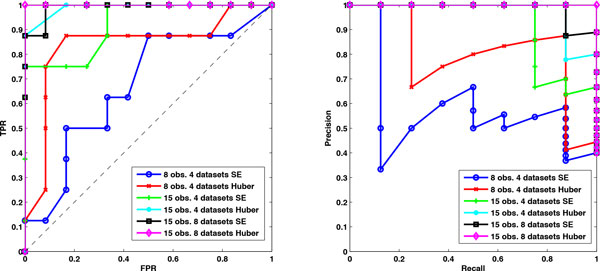
**ROC plots and precision-recall plots of the Huber group LASSO and group LASSO for the simulation data under different situations**. Left: the ROC plots of the Huber group LASSO and the group LASSO. Right: precision-recall plots of the Huber group LASSO and the group LASSO. TPR: true positive rate. FPR: false positive rate. Huber group LASSO has better performance than group LASSO. The larger the number of observations or datasets, the better the performances of the methods.

Data are generated under three situations (*m *= 8*, n_k _*= 15), (*m *= 4*, n_k _*= 15) and (*m *= 4*, n_k _*= 8). Using the stability selection procedure that is introduced in the Method Section, network typologies consisting of edges with scores or probabilities are inferred by Huber group LASSO and group LASSO. For the first two situations, we set the number of bootstrap samples as 30 and the moving block length as 10. For the third situation, we set the number of bootstrap samples as 30 and the moving block length as 5. Varying the threshold, the ROC plots and precision-recall plots of each method for different situations are obtained and illustrated in Figure 1. The areas under the ROCs (AUROCs) and precision-recall curves (AUPRs) are calculated and reported in Table 1. From Figure 1 and Table 1 we can see that for each situation, the Huber group LASSO outperforms the group LASSO, i.e. the AUROC and AUPR of Huber group LASSO are larger than those of group LASSO. ROC plots in Figure 1 also show that both methods have better performances than the random guess. For the case of *m *= 8 and *n_k _*= 15, the Huber group LASSO even achieves the maximum value of AUROC and AUPR. It can also be seen that for each method, the more the observations or the more the datasets, the larger AUROC and AUPR can be obtained. This is in accord with the intuition because, in this example, more observations or datasets indicate more information as these simulated data are generated under quite similar circumstances. All the simulation results have demonstrated the effectiveness of our proposed method.

**Table 1:** The areas under ROC (AUROC) and precision-recall (AUPR) of the Huber group LASSO and the group LASSO for the simulation datasets under different situations.

Situation	Method	AUROC	AUPR
15 observations 8 datasets	SE	0.9896	0.9852
	Huber	1.0000	1.0000

15 observations 4 datasets	SE	0.9219	0.9169
	Huber	0.9896	0.9736

8 observations 4 datasets	SE	0.6719	0.5749
	Huber	0.8385	0.8049

SE: group LASSO. Huber: Huber group LASSO.

### *In vivo *reverse engineering and modeling assessment (IRMA) data

The data used in this example come from the *In vivo *Reverse Engineering and Modeling Assessment (IRMA) experiment [17], where a network composed of five genes (GAL80, GAL4, CBF1, ASH1 and SWI5) was synthesized in yeast *Saccharomyces cerevisiae*, in which genes regulate each other through a variety of regulatory interactions. The network is negligibly affected by endogenous genes and it is responsive to small molecules. Galactose and glucose are respectively used to switch on and off the network. In this study, we use the IRMA time-course data consisting of four switch off datasets (with the number of time points varying from 19 to 21) and five switch on datasets (with the number of time points varying from 11 to 16).

**Figure 2:**
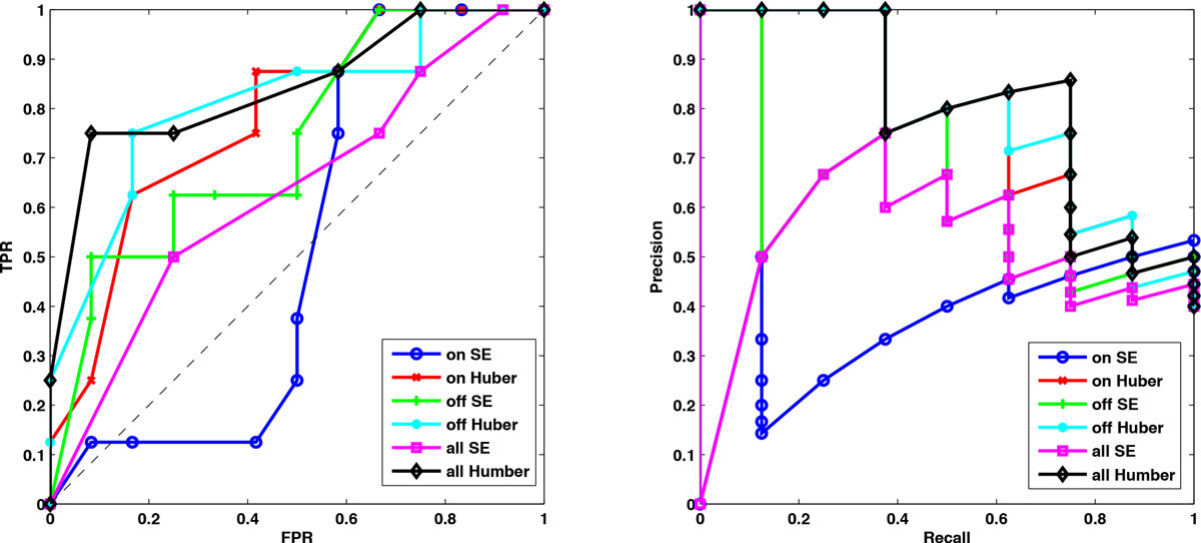
**ROC plots and precision-recall plots of the Huber group LASSO and group LASSO for the IRMA datasets **. Left: the ROC plots of the Huber group LASSO and the group LASSO. Right: precision-recall plots of the Huber group LASSO and the group LASSO. TPR: True positive rate. FPR: false positive rate. Huber group LASSO has better performance than group LASSO.

**Figure 3:**
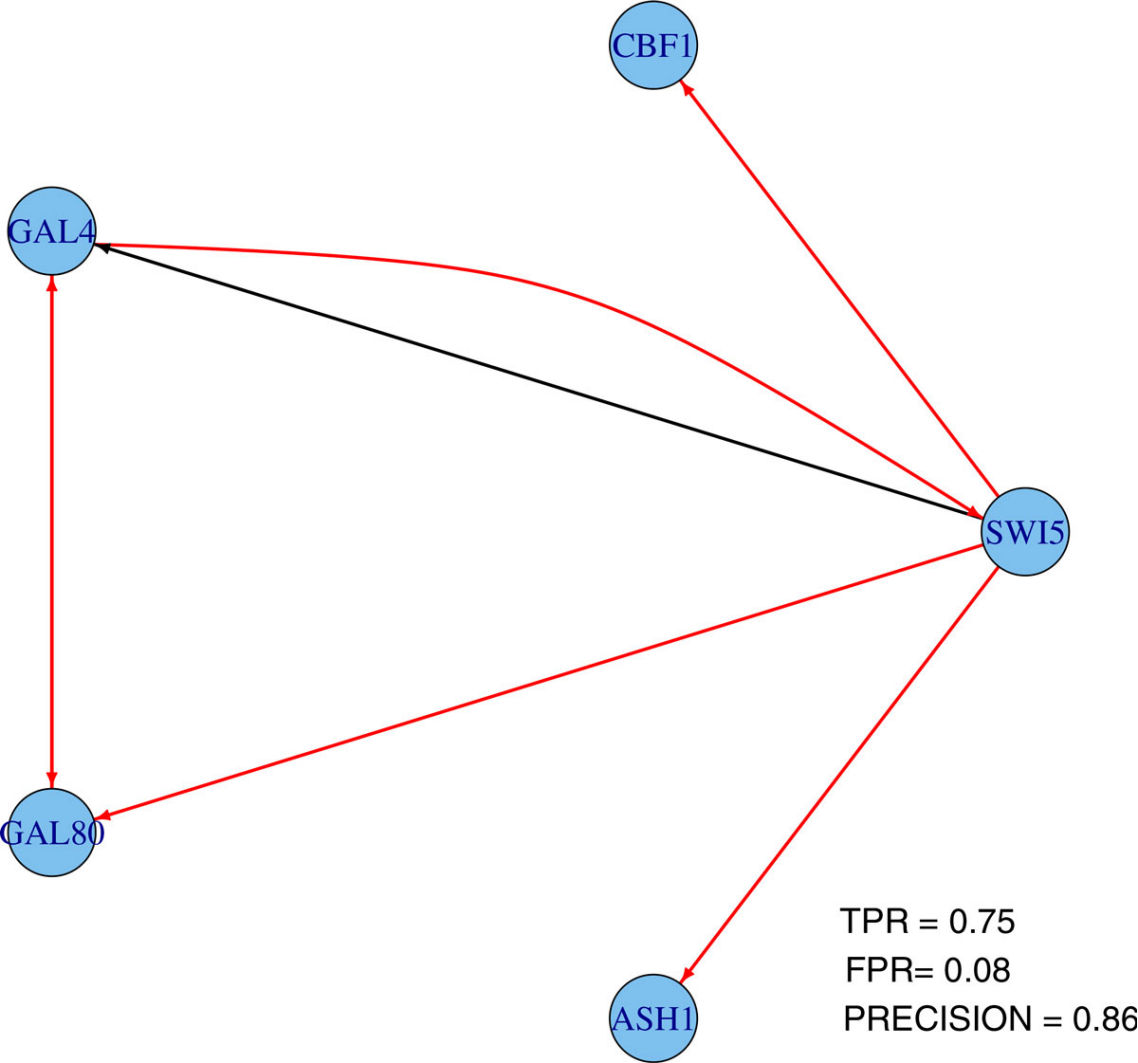
**One network topology from Huber group LASSO using all IRMA datasets**. TPR: true positive rate. FPR: false positive rate.

The Huber group LASSO and the group LASSO are applied to these data in three cases: (1) switch on datasets, (2) switch off datasets and (3) all datasets, i.e., combing switch on and switch off datasets. In the stability selection procedure, the number of bootstrap samples is 30 for all cases and the moving block length is 14 for the second case and 8 for the other cases. The ROC plots and precision-recall plots for the Huber group LASSO and the group LASSO for each case are illustrated in Figure 2 and the corresponding AUROCs and AUPRs are summarized in Table 2. It can be seen that except the group LASSO for the switch on datasets, the performances of the methods are better than random guesses. The Huber group LASSO outperforms the group LASSO in both AUROCs and AUPRs. All methods for the switch off datasets perform better than for the switch on datasets. The group LASSO for all datasets has better performance than for the switch on datasets but is not as good as for the switch off datasets. The Huber group LASSO for all datasets has the best performance among all cases. This indicates that combing multiple datasets may lead to either the best result or a robust result which is better than the worst case. The network topology with false positive rate (FPR) 0.08 of the Huber group LASSO for all datasets is shown in Figure 3 and the corresponding true positive rate (TPR) is 0.75 with precision 0.86, in which the red edges represent true positives while black edges are false positives. The results show the effectiveness of our method for the IRMA data.

**Table 2:** The areas under ROC (AUROC) and precision-recall (AUPR) of the Huber group LASSO and the group LASSO for the IRMA datasets.

Case	Method	AUROC	AUPR
Switch on datasets	SE	0.5208	0.3711
	Huber	0.7812	0.6971

Switch off datasets	SE	0.7344	0.6341
	Huber	0.8125	0.7928

All datasets	SE	0.6302	0.5122
	Huber	0.8438	0.8049

SE: group LASSO. Huber: Huber group LASSO.

### *E. coli *SOS network

**Figure 4:**
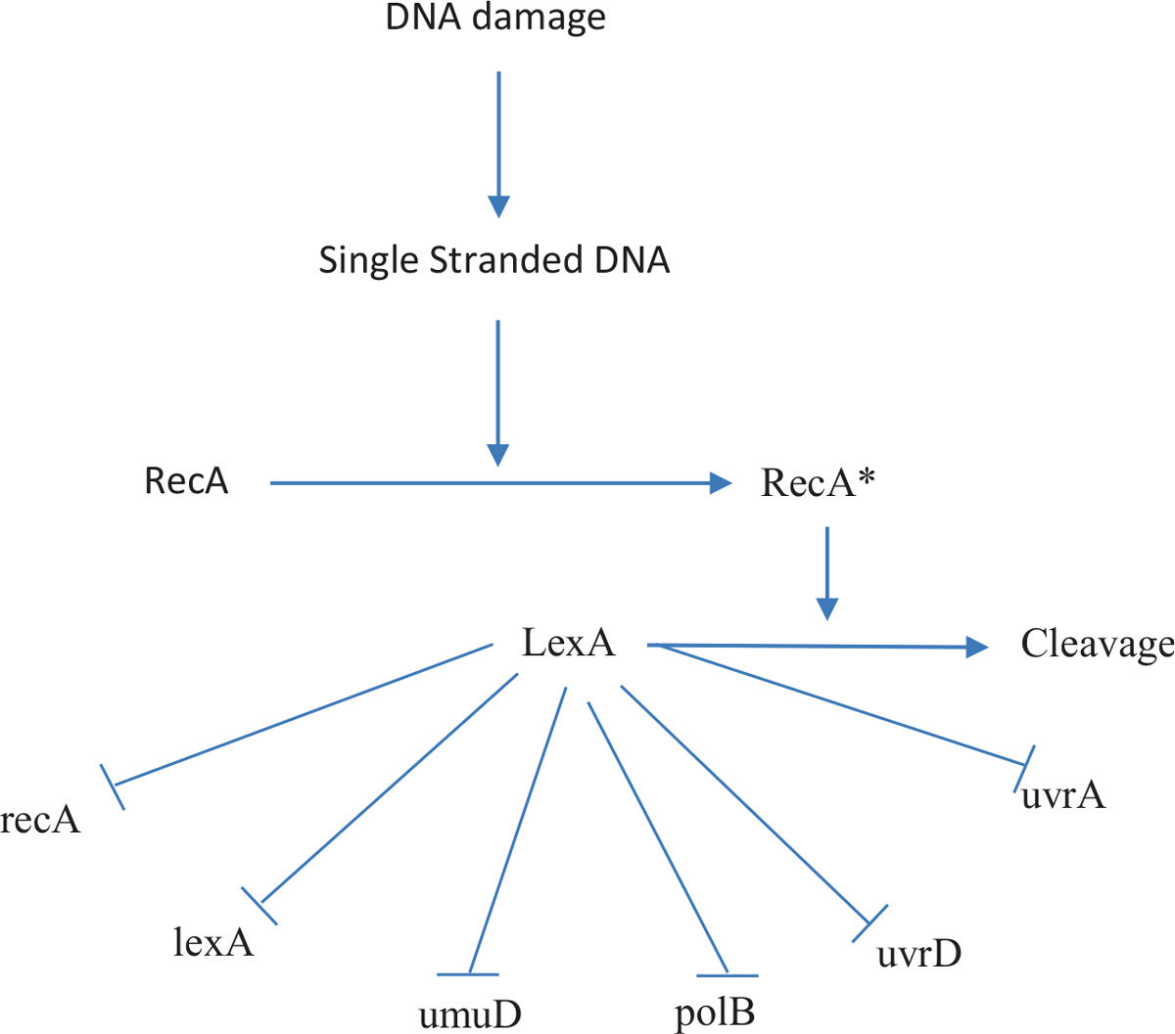
**SOS DNA repair pathway in E**. coli. The arrow represent activation while the flat arrow represents inhibition. Genes are in lower cases, proteins in capital letters.

In this example, we apply the proposed method to identify the real GRN, *E. coli *SOS DNA repair system as shown in Figure 4. This network is responsible for repairing the DNA after some damage happens. LexA acts as the master repressor of many genes in the normal states. When a damage occurs, RecA acts as a sensor and binds to single-stranded DNA to sense the damage and mediates the autocleavage of LexA. The repressions of the SOS genes are halted by the drop in LexA levels. The SOS genes are activated and start to repair the damages. When the repair is done, RecA level drops and stops mediating the autocleavage of LexA. Then, LexA accumulates and represses the SOS genes to make the cell go back to the normal state.

Four time-course gene expression datasets of SOS DNA network are downloaded from the Uri Alon lab (http://www.weizmann.ac.li/mcb/UriAlon.), which are produced from four experiments for various UV light intensities (Experiment 1 and 2: 5 *J m^−^*2, Experiment 3 and 4: 20 *J m^−^*2). Each dataset contain 8 genes and their measurements at 50 time points. As other literature did, e.g. [18-21], only 6 genes, i.e., uvrD, lexA, umuD, recA, uvrA and polB are considered because they are well studied and the gold standard network of these genes are illustrated in Table 3. Details of the gold standard can be found in [18]. In this study, we do not consider the signs and the self-regulations.

**Table 3:** Gold standard of SOS network, collected from literature [18].

	uvrD	lexA	umuD	recA	uvrA	polB
uvrD	0	-1	-1	1	1	0
lexA	0	-1	-1	1	0	0
umuD	0	-1	-1	1	0	0
recA	0	-1	-1	1	0	0
uvrA	1	-1	-1	1	0	0
polB	0	-1	-1	1	0	0

**Figure 5:**
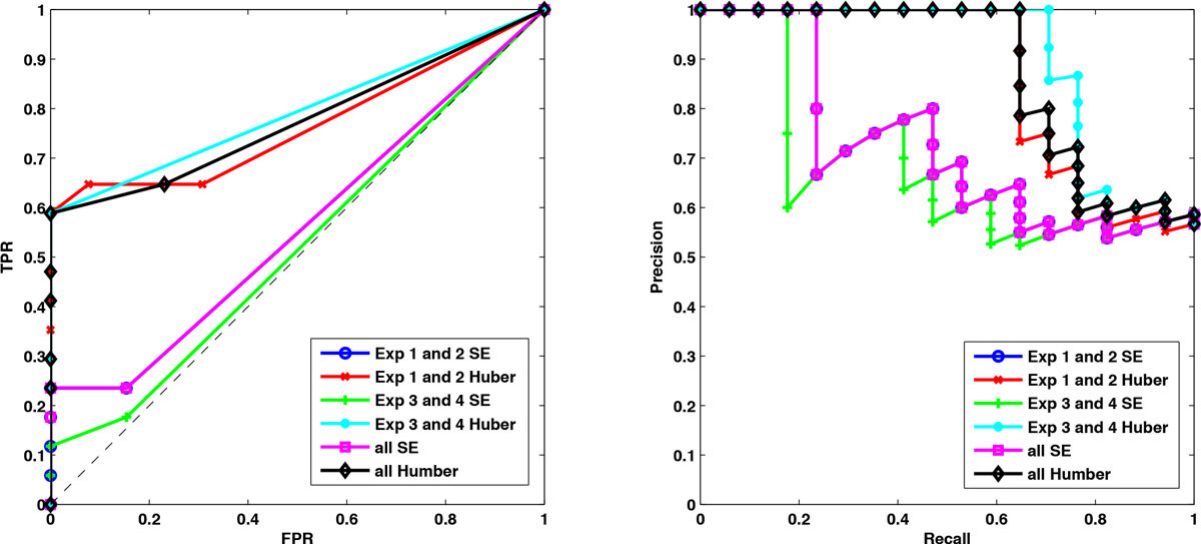
**ROC plots and precision-recall plots of the Huber group LASSO and group LASSO for E**. coli SOS datasets. Left: the ROC plots of the Huber group LASSO and the group LASSO. Right: precision-recall plots of the Huber group LASSO and the group LASSO. TPR: true positive rate. FPR: false positive rate. Huber group LASSO has better performance than group LASSO.

**Figure 6:**
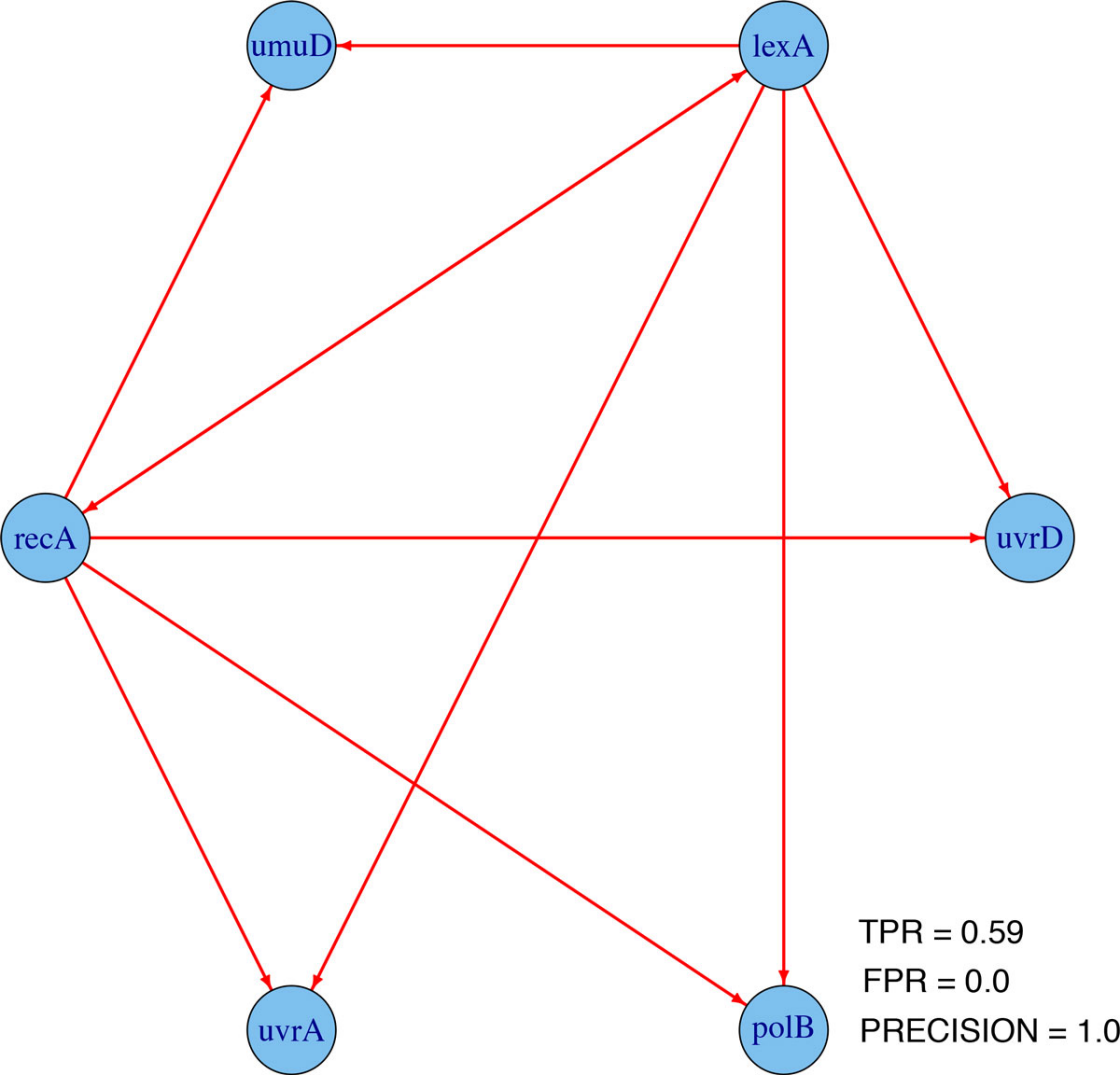
**One network topology from Huber group LASSO using all E**. coli SOS datasets. TPR: true positive rate. FPR: false positive rate.

As the conditions for the first two experiments are different for the last two experiments, we consider applying the method to three cases: (1) datasets of experiment 1 and 2, (2) datasets of experiment 3 and 4 and (3) all experiment datasets. In the stability selection procedure, the number of bootstrap samples is 30 and the moving block length is 25 for all cases. The ROC plots and precision-recall plots for the Huber group LASSO and the group LASSO for each case are illustrated in Figure 5 and the corresponding AUROCs and AUPRs are illustrated in Table 4. From the ROC plots and AUROCs, it can be seen that the Huber group LASSO performs significantly better than random guess while the group LASSO method is only a little bit better than random guess. Obviously, the Huber group LASSO outperforms the group LASSO both in AUROCs and AUPR for all cases. The Huber group LASSO using experiment 3 and 4 datasets has the best performance. Performance of the Huber group LASSO using all datasets is between that in the first case and that in the second case. It can be considered as a robust result because of the using of multiple datasets. The network topology with FPR 0 and TPR 0.59 of the Huber group LASSO for all datasets is shown in Figure 6, in which all inferred edges are correct. These results demonstrate the effectiveness of our method for the *E. coli *SOS data.

**Table 4:** The areas under ROC (AUROC) and precision-recall (AUPR) of the Huber group LASSO and the group LASSO for the E.

Case	Method	AUROC	AUPR
Experiment 1 and 2	SE	0.5588	0.7225
	Huber	0.7670	0.8649

Experiment 3 and 4	SE	0.5204	0.6801
	Huber	0.7941	0.8981

All experiment data	SE	0.5588	0.7225
	Huber	0.7760	0.8756

coli SOS datasets. SE: group LASSO. Huber: Huber group LASSO.

### S. cerevisae cell cycle subnetwork

A cell cycle regulatory subnetwork in S. cerevisae is inferred by the proposed method from 5 experimental microarray datasets. As in [22], the subnetwork consists of 27 genes including 10 genes for producing transcription factors (ace2, fkh1, swi4, swi5, mbp1, swi6, mcm1, fkh2, ndd1, yox1) and 17 genes for producing cyclin and cyclin/CDK regulatory proteins (cln1, cln2, cln3, cdc20, clb1, clb2, clb4, clb5, clb6, sic1, far1, spo12, apc1, tem1, gin4, swe1 and whi5). The time-course datasets we use include cell-cycle alpha factor release, cdc15, alpha factor fkh1 fkh2, fkh1,2 alpha factor and Elutriation, which are all downloaded from Stanford Microarray Database (SMD). We apply the Huber group LASSO and the group LASSO respectively to infer the network from the datasets.

**Figure 7:**
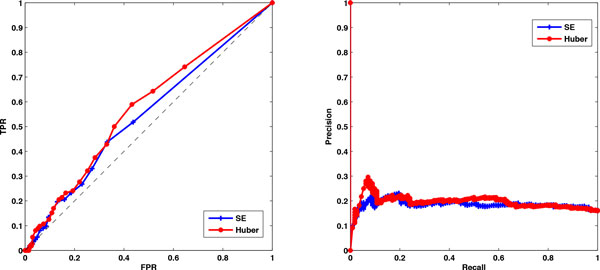
**ROC plots and precision-recall plots of the Huber group LASSO and group LASSO for the cell cycle datasets**. Left: the ROC plots of the Huber group LASSO and the group LASSO. Right: precision-recall plots of the Huber group LASSO and the group LASSO. TPR: true positive rate. FPR: false positive rate. Huber group LASSO has better performance than group LASSO.

**Figure 8:**
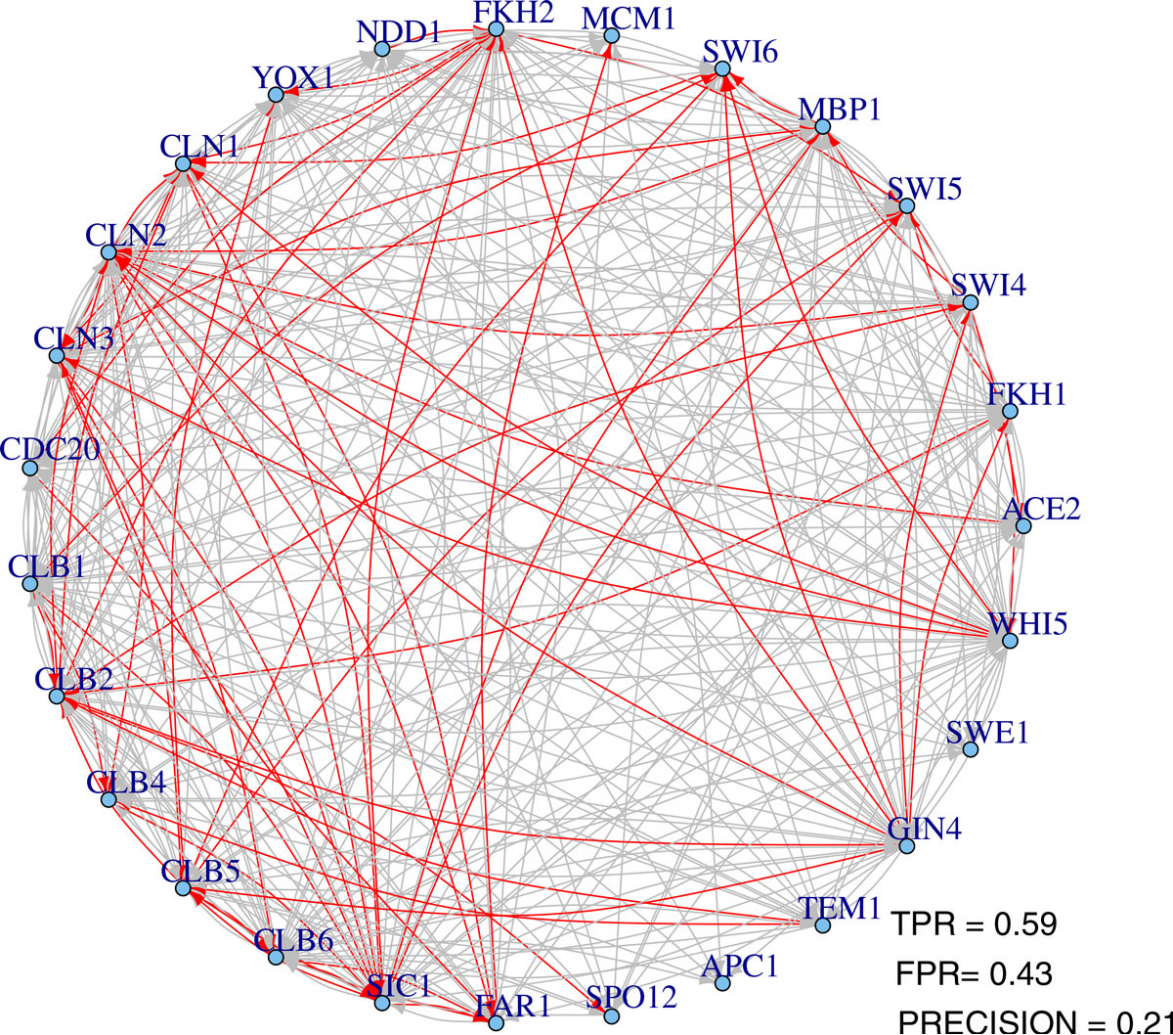
**One network topology from Huber group LASSO for cell cycle datasets**. TPR: true positive rate. FPR: false positive rate.

In order to demonstrate the effectiveness of the proposed method, the inferred results are compared with the interaction network of the chosen 27 genes, drawn from BioGRID database [23]. The network in the database has 112 interactions, not including the self-regulations, and we take it as the gold standard regulatory network. In the stability selection procedure, the number of bootstrap samples is 30 and the moving block length is 9. The ROC plots and precision-recall plots are illustrated in Figure 7 and the AUROCs and AUPRs are shown in Table 5. We can see that both methods have better performances than random guess and the Huber group LASSO outperforms the group LASSO. One network from Huber group LASSO with FPR 0.43 and TPR 0.59 is shown in Figure 8, in which red edges are those inferred edges having been identified in the database and grey edges might be either false positives or novel discovered regulatory relations. Although the gold standard network extracted from the database may contain false edges or not be complete, this shows the effectiveness of our method to some extent.

**Table 5:** The areas under ROC (AUROC) and precision-recall (AUPR) of the Huber group LASSO and the group LASSO for the cell cycle datasets.

Method	AUROC	AUPR
SE	0.5466	0.1844
Huber	0.5753	0.1941

SE: group LASSO. Huber: Huber group LASSO.

## Conclusions

A novel method, Huber group LASSO, has been proposed to integrate multiple time-course gene expression datasets to infer the underlying GRN topology. As an extension to the group LASSO, it is robust to large noises and outliers. An efficient algorithm which combines the ideas of auxiliary function minimization and block descent is developed to slove the involved optimization problem. The convergence analysis of the algorithm shows that the sequence generated from the algorithm indeed converges to the optimal solution of the problem. Instead of selecting a specific tuning parameter corresponding to a determinant network topology, an adapted stability selection procedure is used to lead to a network consisting of edges with scores. The applications of the proposed method to the simulation datasets and real experimental datasets show that Huber group LASSO outperforms the group LASSO in both AUROC and AUPR. It also shows that the integration of multiple time-course gene expression datasets by the proposed method lead to reliable inferred network typologies.

The information in the gene expression data is quite limited. One direction of the future work along this study is to extend the method to be able to integrate other types of data with the gene expression data.

## Method

### Huber group LASSO

Given *m *datasets, $\tilde{X}\left(1\right),\ldots ,\tilde{X}\left(m\right)$, satisfying model (5), to ensure that all **A**(*k*)'s have the same structure, elements of **A**(*k*)'s on the same position are grouped together and can be inferred by the group LASSO,

$$\underset{A\left(k\right)}{\text{min}}\overset{p}{\underset{i=1}{\sum }}\overset{m}{\underset{k=1}{\sum }}w_{k}\overset{n_{k}}{\underset{j=1}{\sum }}{\left(y_{ij}\left(k\right)-A_{i}{\left(k\right)}^{T}x_{j}\left(k\right)\right)}^{2}+\lambda \overset{p}{\underset{i=1}{\sum }}\overset{p}{\underset{\ell =1}{\sum }}\sqrt{a_{i\ell }{\left(1\right)}^{2}+\ldots +a_{i\ell }{\left(m\right)}^{2}},$$

where **A***_i_*(*k*)*^T ^*is the *i*th row of the matrix **A**(*k*) and **x***_j _*(*k*) is the *j*th column of the matrix **X**(*k*). *w_k _*is the weight for the *k*th dataset, which can be assigned by experience. In this study, we choose *w_k _*= *n_k _/*Σ*n_k _*i.e., the more observations the dataset has, the higher weight it is assigned with. The penalty term in (6) takes advantage of the sparse nature of GRNs and has the effect making the grouped parameters to be estimated either all zeros or all non-zeros [10], i.e., *a_iℓ_*(*k*)'s, *k *= 1*, . . . , m*, become either all zeros or all non-zeros. Therefore, a consistent network topology can be obtained from the group LASSO method. *λ *is a tuning parameter which controls the degree of sparseness of the inferred network. The larger the value of *λ*, the more grouped parameters become zeros.

To introduce robustness, we consider using the Huber loss function instead of the squared error loss function and propose the following Huber group LASSO method

(7) $$\underset{A\left(k\right)}{\text{min}}\overset{p}{\underset{i=1}{\sum }}\overset{m}{\underset{k=1}{\sum }}w_{k}\overset{n_{k}}{\underset{j=1}{\sum }}H_{\delta }\left(y_{ij}\left(k\right)-A_{i}{\left(k\right)}^{T}x_{j}\left(k\right)\right)+\lambda \overset{p}{\underset{i=1}{\sum }}\overset{p}{\underset{\ell =1}{\sum }}\sqrt{a_{i\ell }{\left(1\right)}^{2}+\ldots +a_{i\ell }{\left(m\right)}^{2}},$$

where the Huber loss function is defined as

(8) $$H_{\delta }\left(\theta \right)=\left\{\begin{array}{cc} \theta^{2} & \text{if}\;|\theta |\leq \delta \\ 2\delta |\theta |-\delta^{2} & \text{otherwise}. \end{array}\right)$$

**Figure 9:**
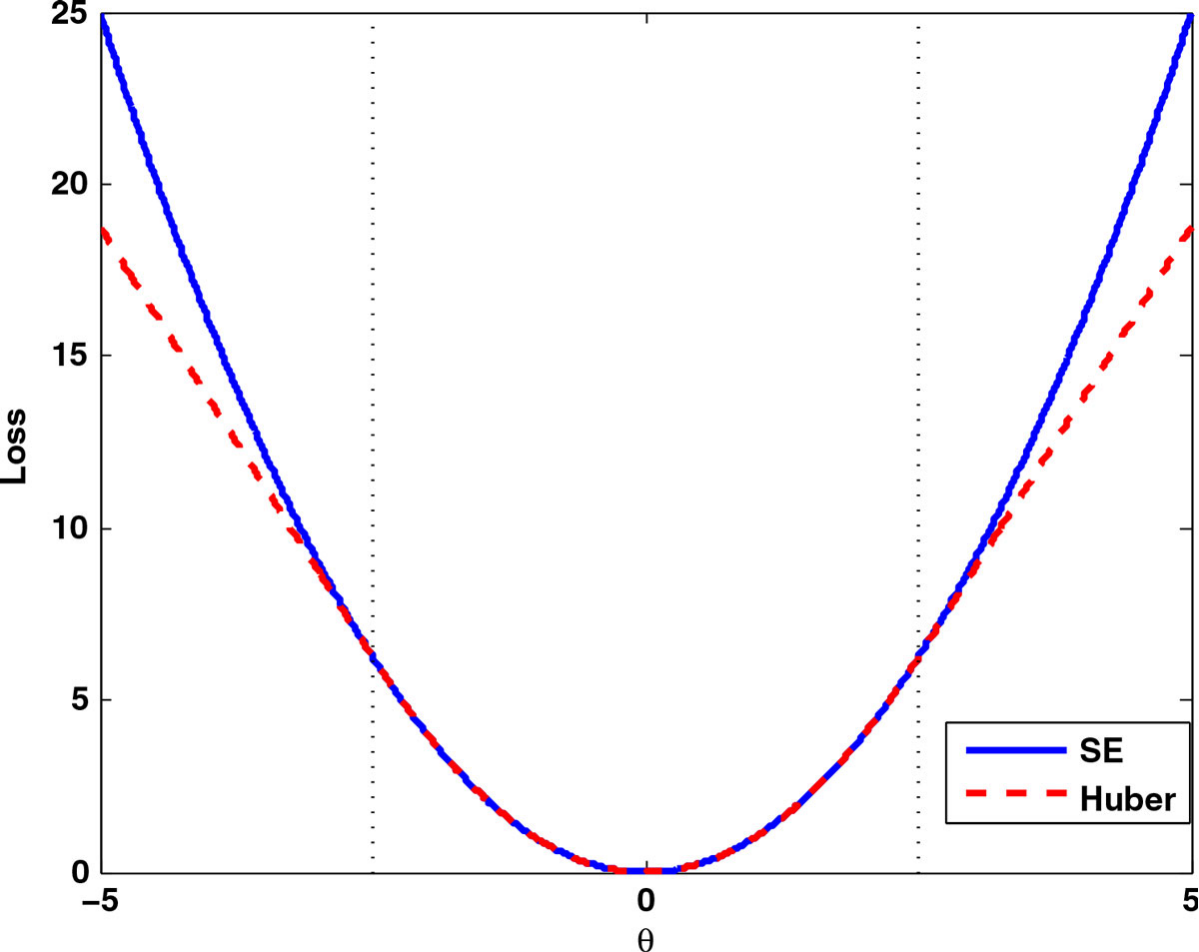
**Squared error and Huber loss functions**. For small error, *θ*, squared error loss and Huber loss are the same. For large error, squared error penalizes quadratically while Huber loss penalizes linearly.

The squared error and Huber loss function are illustrated in Figure 9. It can be seen that for small errors, these two loss functions are exactly the same while for large errors, Huber loss which increases linearly is less than the squared error loss which increases quadratically. Because Huber loss penalizes much less than the squared error loss for large errors, the Huber group LASSO is more robust than group LASSO when there exists large noise or outliers in the data. It is also known that the Huber loss is nearly as efficient as squared error loss for Gaussian errors [24].

For convenience, we define some notations and rewrite the problems (6) and (7) in more compact forms. Let **Y***_i _*= [**Y***_i_*(1)*^T ^, . . . *, **Y***_i_*(*m*)*^T^*]*^T^*, the vector stacking observations of the *i*th target gene across all datasets, where **Y***_i_*(*k*)*^T ^*is the *i*th row of **Y**(*k*). Let **b***_iℓ_*= [*a_iℓ_*(1)*, . . . , a_iℓ_*(*m*)]*^T^*, the vector containing the grouped parameters. Denote by $b_{i}={\left[b_{i1}^{T},\ldots ,b_{ip}^{T}\right]}^{T}$ the vector containing all parameters related to the regulation of the *i*th target gene. According to the orders of the parameters in **b***_i_*, re-arrange the rows of **X**(*k*) and piece them together to have $X={\left[X_{1}^{T},\ldots ,X_{p}^{T}\right]}^{T}$ where **X***_i _*= diag(**X***_i_*(1)*^T ^, . . . *, **X***_i_*(*m*)*^T^*) with **X***_i_*(*k*)*^T ^*being the *i*th row of **X**(*k*). Then (7) can be rewritten as

(9) $$\overset{p}{\underset{i=1}{\sum }}\overset{n}{\underset{j=1}{\sum }}\omega_{j}H_{\delta }\left(y_{ij}-x_{j}^{T}b_{i}\right)+\lambda \overset{p}{\underset{i=1}{\sum }}\overset{n}{\underset{\ell =1}{\sum }}{∥b_{i\ell }∥}_{2},$$

where **x***_j _*is the *j*th column of **X**, *y_ij _*is the *j*th element of **Y***_i_*, $n=\sum_{k=1}^{m}n_{k}$ and $\omega_{i}=w_{1}I\left(i\leq n_{1}\right)+\sum_{k=2}^{m}w_{k}I\left(\sum_{l=1}^{k=1}n_{l}<i\leq \sum_{l=1}^{k}n_{l}\right)$. (6) can be rewritten similarly.

### Optimization algorithm

The minimization of problem (9) is not easy as the penalty term is not differentiable at zero and the Huber loss does not have the second order derivatives at the transition points, *±δ*. Observed that fixing *i*, the problem (9) can be decomposed into *p *sub-optimization problems. For each, we get **b***_i _*by minimizing

(10) $$J\left(b\right)=\overset{n}{\underset{j=1}{\sum }}\omega_{j}H_{\delta }\left(y_{j}-x_{j}^{T}b_{i}\right)+\lambda \overset{n}{\underset{\ell =1}{\sum }}{∥b_{\ell }∥}_{2},$$

where for notational convenience, we omit the subscript *i *here and **b***_ℓ _*is a block of parameters of **b**, i.e. $b={\left[b_{1}^{T},\ldots ,b_{p}^{T}\right]}^{T}$.

To optimize (10), an iterative method is developed by constructing an auxiliary function, the optimization of which keeps *J *(**b**) decreasing. As in [13], given any current estimate **b**^(*k*)^, a function *Q*(**b***|***b**^(*k*)^) is an auxiliary function for *J *(**b**) if conditions

(11) $$J\left(b^{\left(k\right)}\right)=Q\left(b^{\left(k\right)}|b^{\left(k\right)}\right)\;\;\;and\;\;\;J\left(b\right)\leq Q\left(b|b^{\left(k\right)}\right)\;\;\;\text{for}\;\text{all}\;b,$$

are satisfied. In this study, we construct the auxiliary function as

(12) $$Q\left(b|b^{\left(k\right)}\right)=\overset{n}{\underset{j=1}{\sum }}\omega_{j}H_{\delta }\left(y_{j}-x_{j}^{T}b^{\left(k\right)}\right)-\overset{n}{\underset{j=1}{\sum }}\omega_{j}{H^{\prime }}_{\delta }\left(y_{j}-x_{j}^{T}b^{\left(k\right)}\right)x_{j}^{T}\left(b-b^{\left(k\right)}\right)+2\gamma {∥b-b^{\left(k\right)}∥}_{2}^{2}+\lambda \overset{p}{\underset{\ell =1}{\sum }}{∥b_{\ell }∥}_{2},$$

where *γ *is the largest eigenvalue of $\sum_{j=1}^{n}\omega_{j}x_{j}x_{j}^{T}$. It can be easily shown that this auxiliary function satisfies (11).

Considering the block structure of **b**, we apply a block-wise descent strategy [14], i.e., cyclically optimize one block of parameters, **b***_j _*, at a time. Denote by $b^{\left(k\right)}\left(\ell \right)={\left[b_{1}^{{\left(k+1\right)}^{T}},\ldots ,b_{\ell }^{{\left(k+1\right)}^{T}},b_{\ell +1}^{{\left(k\right)}^{T}},\ldots ,b_{p}^{{\left(k\right)}^{T}}\right]}^{T}$ the vector after updating the *R*th block. Given **b**^(*k*)^(*ℓ− *1), update it to **b**^(*k*)^(*ℓ*) by computing

(13) $$\begin{array}{c} b_{\ell }^{\left(k+1\right)}=\underset{b_{\ell }}{\text{arg}\;\text{min}}\;Q\left({\left[b_{1}^{{\left(k+1\right)}^{T}},\ldots ,b_{\ell -1}^{{\left(k+1\right)}^{T}},b_{\ell }^{T},b_{\ell +1}^{{\left(k\right)}^{T}},\ldots ,b_{p}^{{\left(k\right)}^{T}}\right]}^{T}|b^{\left(k\right)}\left(\ell -1\right)\right)\\ =\left(\frac{1}{4\gamma }-\frac{\lambda }{4\gamma {∥\sum_{j=1}^{n}\omega_{j}{H^{\prime }}_{\delta }\left(y_{j}-x_{j}^{T}b^{\left(k\right)}\left(\ell -1\right)\right)x_{j,\ell }+4\gamma b_{\ell }^{\left(k\right)}∥}_{2}}\right)\\ \times \left(\overset{n}{\underset{j=1}{\sum }}\omega_{j}{H^{\prime }}_{\delta }\left(y_{j}-x_{j}^{T}b^{\left(k\right)}\left(\ell -1\right)\right)x_{j,\ell }+4\gamma b_{\ell }^{\left(k\right)}\right). \end{array}$$

where **x***_j,ℓ _*is the block of elements in **x***_j _*corresponding to **b***_ℓ _*and (*·*)_+ _= max(*·*, 0). We repeat to update every block using (13) until it converges. For a specific value of *λ*, the whole procedure is described as follows:

1 Initialize **b**(0). Set iteration number *k *= 0.

2 Cycle through (13) one at a time to update the *ℓ*th block, *ℓ *= 1*, . . . , p*

3 If {**b**^(*k*)^} converges to **b**^∗^, go to the next step. Otherwise, set *k *:= *k *+ 1 and go to Step 2.

4 Return the solution **b**^∗^.

Note that the algorithm can be adapted to solve (6) with quite similar derivations. In the following section, we show that the sequence {**b**^(*k*)^} generated from the algorithm guarantees the objective function *J *(**b**) keep decreasing. We also show that the limit point of the sequence generated is indeed the minimum point of *J *(**b**).

### Convergence analysis

The convergence of the optimization algorithm for the minimization of (10) is analyzed in the way similar to [25]. We first show the descent property of the algorithm.

**Lemma 1 ***The sequence *{**b**(k)} *generated from the optimization algorithm keeps the objective function *J(**b**) *decreasing, i.e., *J(**b**^(k)^) ≥ J (**b**^(k+1)^).

*Proof *By (11) and (13), we have

$\begin{array}{cc} J\left(b^{\left(k\right)}\right) & =Q\left(b^{\left(k\right)}|b^{\left(k\right)}\right)\geq Q\left(b^{\left(k\right)}\left(1\right)|b^{\left(k\right)}\right)\\ & \geq Q\left(b^{\left(k\right)}\left(2\right)|b^{\left(k\right)}\left(1\right)\right)\geq \cdots \geq Q\left(b^{\left(k\right)}\left(p\right)|b^{\left(k\right)}\left(p-1\right)\right)\geq J\left(b^{\left(k\right)}\left(p\right)\right)\\ & =J\left(b^{\left(k+1\right)}\right). \end{array}$

Next, we show that if the generated sequence satisfies some conditions, it converges to the optimal solution.

**Lemma 2 ***Assume the data *(**y**, **X**) *lies on a compact set and the following conditions are satisfied:*

*1 The sequence *{**b**^(k)^} *is bounded*.

*2 For every convergent subsequence *$\left\{b^{\left(n_{k}\right)}\right\}\subset \left\{b^{\left(k\right)}\right\}$, the successive differences converge to zeros, $b^{\left(n_{k}\right)}-b^{\left(n_{k}-1\right)}\to 0$.

*Then, every limit point ***b**^∞ ^*of the sequence *{**b**^(k)^} *is a minimum for the function *J(**b**), *i.e., for any *$\delta ={\left(\delta_{1}^{T},\ldots ,\delta_{p}^{T}\right)}^{T}\in {\mathbb{R}}^{mp}$,

*Proof *For any $b={\left(b_{1}^{T},\ldots ,b_{p}^{T}\right)}^{T}\in {\mathbb{R}}^{mp}$ and $\delta \left(j\right)={\left(0^{T},\ldots ,\delta_{j}^{T},\ldots ,0^{T}\right)}^{T}\in {\mathbb{R}}^{mp}$

$$\underset{\alpha ↓0+}{\text{lim}\text{inf}}\left\{\frac{J\left(b+\alpha \delta \left(j\right)\right)-J\left(b\right)}{\alpha }\right\}=\nabla_{j}f{\left(b\right)}^{T}\delta_{j}+\underset{\alpha ↓0+}{\text{lim}\text{inf}}\left\{\frac{\lambda {∥b_{j}+\alpha \delta_{j}∥}_{2}-{∥b_{j}∥}_{2}}{\alpha }\right\},$$

where $f\left(b\right)=\sum_{i=1}^{n}\omega_{i}H_{\delta }\left(y_{i}-x_{i}^{T}b\right)$ and ∇*_j _*represents the partial derivatives with respect to the *j*th block of parameters. Denote the second term by *∂P *(**b***_j _*; ***δ***_j _) and it has

(14) $$\partial P\left(b_{j};\delta_{j}\right)=\left\{\begin{array}{cc} \lambda \frac{b_{j}^{T}\delta_{j}}{{∥b_{j}∥}_{2}} & \text{if}\;\;b_{j}\neq 0,\\ \lambda {∥\delta_{j}∥}_{2} & \text{otherwise}\text{.} \end{array}\right)$$

We assume the subsequence $\left\{b^{\left(n_{k}\right)}\right\}$ converges to $b^{\infty }={\left(b_{1}^{\infty T},\ldots ,b_{p}^{\infty T}\right)}^{T}\in {\mathbb{R}}^{mp}$. From condition 2. and (14), we have

$$b^{\left(n_{k}-1\right)}\left(j\right)={\left(b_{1}^{{\left(n_{k}\right)}^{T}},\ldots ,b_{j}^{{\left(n_{k}\right)}^{T}},b_{j+1}^{{\left(n_{k}-1\right)}^{T}},\ldots ,b_{p}^{{\left(n_{k}-1\right)}^{T}}\right)}^{T}\to b^{\infty },\;\;\;\;\text{as}\;k\to \infty ,$$

and

(15) $$\text{if}\;b_{j}^{\infty }\neq 0,\;\;\;\;\partial P\left(b_{j}^{\left(n_{k}\right)};\delta_{j}\right)\to \partial P\left(b_{j}^{\infty };\delta_{j}\right);\;\;\;\;\text{if}\;b_{j}^{\infty }=0,\;\;\;\;\;\;\partial P\left(b_{j}^{\infty };\delta_{j}\right)\geq \underset{k\to \infty }{\text{lim}\;\text{inf}}\partial P\left(b_{j}^{\left(n_{k}\right)};\delta_{j}\right),$$

since $b_{j}^{T}\delta_{j}\leq {∥b_{j}∥}_{2}{∥\delta_{j}∥}_{2}$.

As $b_{j}^{\left(n_{k}\right)}$ minimizes $Q\left({\left(b_{1}^{{\left(n_{k}\right)}^{T}},\ldots ,b_{j-1}^{{\left(n_{k}\right)}^{T}},b_{j}^{T},b_{j+1}^{{\left(n_{k}-1\right)}^{T}},\ldots ,b_{p}^{{\left(n_{k}-1\right)}^{T}}\right)}^{T}|b^{\left(n_{k}\right)}\left(j-1\right)\right)$ with respect to the *j*th block of parameters, using (14), we have

(16) $$\nabla_{j}q{\left(b^{\left(n_{k}\right)}\left(j\right)|b^{\left(n_{k}\right)}\left(j-1\right)\right)}^{T}\delta_{j}+\partial P\left(b_{j}^{\left(n_{k}\right)};\delta_{j}\right)\geq 0,\;\;\;\text{for}\;\text{all}\;k,$$

with

$$\begin{array}{cc} q\left(b^{\left(n_{k}\right)}\left(j\right)|b^{\left(n_{k}\right)}\left(j-1\right)\right) & =\overset{n}{\underset{i=1}{\sum }}\omega_{i}H_{\delta }\left(y_{i}-x_{i}^{T}b^{\left(n_{k}\right)}\left(j-1\right)\right)\\ & \;\;\;\;\;-\overset{n}{\underset{i=1}{\sum }}\omega_{i}{H^{\prime }}_{\delta }\left(y_{i}-x_{i}^{T}b^{\left(n_{k}\right)}\left(j-1\right)\right)x_{i}^{T}\left(b^{\left(n_{k}\right)}\left(j\right)-b^{\left(n_{k}\right)}\left(j-1\right)\right)\\ & \;\;\;\;\;+2\gamma {∥b^{\left(n_{k}\right)}\left(j\right)-b^{\left(n_{k}\right)}\left(j-1\right)∥}_{2}^{2} \end{array}$$

Due to condition 2.,

(17) $$\nabla_{j}q\left(b^{\left(n_{k}\right)}\left(j\right)|b^{\left(n_{k}\right)}\left(j-1\right)\right)\to \nabla_{j}f\left(b^{\infty }\right)\;\;\;\;\;\;\;as\;\;k\to \infty .$$

Therefore, (15), (16) and (17) yield

(18) $$\nabla_{j}f{\left(b^{\infty }\right)}^{T}\delta_{j}+\partial P\left(b^{\infty };\delta_{j}\right)\geq \underset{k\to \infty }{\text{lim}\;\text{inf}}\left\{\nabla_{j}q(b^{\left(n_{k}\right)}\left(j\right)|{\left(b^{\left(n_{k}\right)}\left(j-1\right)\right)}^{T}\delta_{j}+\partial P\left(b_{j}^{\left(n_{k}\right)};\delta_{j}\right)\right\}\geq 0,$$

for any 1 *≤ j ≤ p*.

For $\delta ={\left(\delta_{1}^{T},\ldots ,\delta_{p}^{T}\right)}^{T}\in {\mathbb{R}}^{mp}$, due to the differentiability of *f *(**b**),

$\begin{array}{cc} \underset{\alpha ↓0+}{\text{lim}\text{inf}}\left\{\frac{J\left(b^{\infty }+\alpha \delta \right)-J\left(b^{\infty }\right)}{\alpha }\right\} & =\overset{p}{\underset{j=1}{\sum }}\nabla_{j}f{\left(b^{\infty }\right)}^{T}\delta_{j}+\overset{p}{\underset{j=1}{\sum }}\underset{\alpha ↓0+}{\text{lim}\text{inf}}\left\{\frac{\lambda {∥b_{j}^{\infty }+\alpha \delta_{j}∥}_{2}-{∥b_{j}^{\infty }∥}_{2}}{\alpha }\right\}\\ & =\overset{p}{\underset{j=1}{\sum }}\left\{\nabla_{j}f{\left(b^{\infty }\right)}^{T}\delta_{j}+\partial P\left(b^{\infty };\delta_{j}\right)\right\}\geq 0. \end{array}$

Finally, we show that the sequence generated from the proposed algorithm satisfies these two conditions.

**Theorem 3 **Assuming the data (**y**, **X**) lies on a compact set and no column of **X **is identically **0**, the sequence {**b**^(k)^} generated from the algorithm converges to the minimum point of the objective function J (**b**).

*Proof *We only need to show that the generated sequence meets the conditions in Lemma 2.

For the sake of notational convenience, for fixed *j *and ${\left(b_{1}^{T},\ldots ,b_{j-1}^{T},b_{j+1}^{T},\ldots ,b_{p}^{T}\right)}^{T}$ define

$$\chi \left(\cdot \right):u\mapsto J\left({\left(b_{1}^{T},\ldots ,b_{j-1}^{T},u^{T},b_{j+1}^{T},\ldots ,b_{p}^{T}\right)}^{T}\right).$$

Let **b**(**u**) be the vector containing **u **as its *j*th block of parameters with other blocks being the fixed values.

Assume **u **+ ***δ ***and **u **represent the values of the *j*th block of parameters before and after the block update, respectively. Hence, as defined in (12), **u **is obtained by minimizing the following function with respect to the *j*th block in the algorithm:

(19) $$\begin{array}{c} \;\;\;Q\left(b\left(u\right)|b\left(u+\delta \right)\right)\\ =f\left(b\left(u+\delta \right)\right)+\nabla_{{}_{j}}f{\left(b\left(u+\delta \right)\right)}^{T}\left(u-\left(u+\delta \right)\right)\\ \;\;\;+2\gamma {∥u-\left(u+\delta \right)∥}_{2}^{2}+\lambda {∥u∥}_{2}+\lambda \underset{\ell \neq j}{\sum }{∥b_{\ell }∥}_{2}. \end{array}$$

where $f\left(b\right)=\sum_{i=1}^{n}\omega_{i}H_{\delta }\left(y_{i}-x_{j}^{T}b\right)$ and $\nabla_{j}f\left(b\right)=\sum_{i=1}^{n}\omega_{i}{H^{\prime }}_{\delta }\left(y_{i}-x_{j}^{T}b\right)x_{i,j}$. Thus, **u **should satisfy

(20) $$\nabla_{j}f\left(b\left(u+\delta \right)\right)-4\gamma \delta +\lambda s=0,$$

where **s **= **u***/||***u||**_2 _if **u **≠ 0; ||**s||**_2 _*≤ *1 if **u **= 0. Then, we have

(21) $$\begin{array}{c} \;\;\;\;\;\chi \left(u+\delta \right)-\chi \left(u\right)\\ =f\left(b\left(u+\delta \right)\right)-f\left(b\left(u\right)\right)+\lambda \left(||\left(u+\delta \right)|{|}_{2}-||u|{|}_{2}\right)\\ =\nabla_{j}f{\left(b\left(u+\delta \right)\right)}^{T}\delta -\nabla_{j}f{\left(b\left(u+\delta \right)\right)}^{T}\delta \left[\nabla_{j}f{\left(b\left(u+\delta \right)\right)}^{T}\delta -4\gamma \delta^{T}\delta +\lambda s^{T}\delta \right]\\ \;\;\;\;+4\gamma \delta^{T}\delta +\lambda \left(||\left(u+\delta \right)|{|}_{2}-||u|{|}_{2}-s^{T}\delta \right)\\ =-\left[\overset{n}{\underset{i=1}{\sum }}\omega_{i}{H^{\prime }}_{\delta }\left(y_{i}-x_{j}^{T}b\left(u+\tau \delta \right)\right)x_{i,j}^{T}\delta -\overset{n}{\underset{i=1}{\sum }}\omega_{i}{H^{\prime }}_{\delta }\left(y_{i}-x_{j}^{T}b\left(u+\delta \right)\right)x_{i,j}^{T}\delta \right]\\ \;\;\;\;+4\gamma \delta^{T}\delta +\lambda \left(||\left(u+\delta \right)|{|}_{2}-||u|{|}_{2}-s^{T}\delta \right)\\ \geq -2\gamma \left(1-\tau \right)||\delta |{|}_{2}^{2}+4\gamma ||\delta |{|}_{2}^{2}\geq 2\gamma ||\delta |{|}_{2}^{2}. \end{array}$$

The second and third equalities are obtained using mean value theorem with *τ *∈ (0, 1) and (20). For the first inequality, the following property of the Huber loss function and the property of subgradient are used.

$$\left(H_{\delta }^{\prime }\left(\theta_{1}\right)-H_{\delta }^{\prime }\left(\theta_{2}\right)\right)\left(\theta_{1}-\theta_{2}\right)\leq 2{\left(\theta_{1}-\theta_{2}\right)}^{2}.$$

The result from (21) gives that

(22) $$J\left(b^{\left(k\right)}\left(j-1\right)\right)-J\left(b^{\left(k\right)}\left(j\right)\right)\geq 2\gamma {∥b_{j}^{\left(k\right)}-b_{j}^{\left(k+1\right)}∥}_{2}^{2}=2\gamma {∥b^{\left(k\right)}\left(j-1\right)-b^{\left(k\right)}\left(j\right)∥}_{2}^{2},$$

where $b^{\left(k\right)}\left(j\right)={\left[b_{1}^{{\left(k+1\right)}^{T}},\ldots ,b_{j}^{{\left(k+1\right)}^{T}},b_{j+1}^{{\left(k\right)}^{T}},\ldots ,b_{p}^{{\left(k\right)}^{T}}\right]}^{T}.$

Using (22) repeatedly across every block, for any *k*, we have

$$J\left(b^{\left(k\right)}\right)-J\left(b^{\left(k+1\right)}\right)\geq 2\gamma {∥b^{\left(k\right)}-b^{\left(k+1\right)}∥}_{2}^{2}.$$

Note that by Lemma 1, {*J *(**b**^(*k*)^)} converges as it keeps decreasing and is bounded from below. The convergence of {*J *(**b**^(*k*)^)} yield the convergence of {**b**^(*k*)^}. Hence, conditions of Lemma 2 hold which imply that the limit of {**b**^(*k*)^} is the minimum point of *J *(**b**).

### Implementation

The tuning parameter *λ *controls the sparseness of the resulted network. A network solution path can be obtained by computing networks on a grid of *λ *values from $\lambda_{\text{max}}=\underset{i,\ell }{\text{max}}∥\sum_{j=1}^{n}\omega_{j}{H^{\prime }}_{\delta }\left(y_{ij}\right)x_{j,\ell }∥$, which is the smallest value that gives the empty network, to a small value, e.g. *λ*_min _= 0.01*λ*_max_. In our previous work [12], BIC criterion is used to pick a specific *λ *value which corresponds to a determinant network topology. A method called "stability selection" recently proposed by Meinshausen and Buhlmann [15] finds a network with probabilities for edges. Stability selection performs the network inference method, e.g. group LASSO, many times, resampling the data in each run and computing the frequencies with which each edge is selected across these runs. It has been used with the linear regression method to infer GRNs from steady-state gene expression data in Haury et al. [26] and has shown perspective effectiveness. In this study, we adapt the stability selection method to finding GRN topology from multiple time-course gene expression datasets. Given a family of *m *time-course gene expression datasets $\tilde{X}\left(k\right)\in R^{p\times n_{k}}$, *k *= 1*, . . . , m*, for a specific *λ *∈ Λ, the stability selection procedure is as follows

1 Use moving block bootstrap to draw *N *bootstrap samples for every dataset to form *N *bootstrap families of multiple time-course datasets, i.e. $\tilde{X}{\left(k\right)}^{*\left(b\right)}{\;\}}_{k=1}^{m}$, *b *= 1, . . . , *N*

2 Use the proposed Huber group LASSO to infer ${\left\{A{\left(k\right)}_{\lambda }^{*\left(b\right)}\right\}}_{k=1}^{m}$ from the *b*th bootstrap family of datasets. Denote by $A_{\lambda }^{*\left(b\right)}$ the network topology shared by ${\left\{A{\left(k\right)}_{\lambda }^{*\left(b\right)}\right\}}_{k=1}^{m}$.

3 Compute the frequencies for each edge (*i, j*), i.e., from the gene *j *to gene *i*, in the network

(24) $$\underset{\lambda }{\prod }\left(i,j\right)=\frac{\#\left\{b:A_{\lambda }^{*\left(b\right)}\left(i,j\right)\neq 0\right\}}{N},$$

where $A_{\lambda }^{*\left(b\right)}\left(i,j\right)$ is the (*i, j*)'s entry of $A_{\lambda }^{*\left(b\right)}$ and #{*·*} is the number of elements in that set.

For a set of *λ ∈ *Λ, the probability of each edge in the inferred network is

(25) $$\prod \left(i,j\right)=\underset{\lambda \in \Lambda }{\text{max}}\underset{\lambda }{\prod }\left(i,j\right)$$

The final network topology can be obtained by setting a threshold, edges with probabilities or scores less than which are considered nonexistent. This study only focus on giving a list of edges with scores. The selection of threshold is not discussed here. The stability selection procedure can also be applied with the group LASSO method (6).

Since the data used are time series data, the moving block bootstrap method is employed in the first step to draw bootstrap samples from each dataset. For a dataset with *n *observations, in the moving block bootstrap with block length *l*, the data is split into *n − l *+ 1 blocks: block *j *consists of observations *j *to *j *+ *l − *1, *j *= 1*, . . . , n − l *+ 1. [*n/b*] blocks are randomly drawn from *n − l *+ 1 blocks with replacement and are aligned in the order they are picked to form a bootstrap sample.

Another tuning parameter *δ *controls the degree of robustness. Generally, it picks $\delta =1.345\hat{\sigma }$ where $\hat{\sigma }$ is the estimated standard deviation of the error and $\hat{\sigma }=MAD/0.6745$, where *MAD *is the median absolute deviation of the residuals. In this study, we use the least absolute deviations (LAD) regression to obtain the residuals. To avoid the overfitting of LAD which leads to a very small *δ*, we choose by $\delta =\text{max}\left(1.345\hat{\sigma },1\right)$.

## Competing interests

The authors declare that they have no competing interests.

## Authors' contributions

FXW and LZL conceived and initialized this study. LZL and FXW designed the algorithms and discussed about the results. LZL implemented the algorithms and performed the experiments, drafted the manuscript while FXW and WJZ modified it. All authors have approved the manuscript.

## Declarations

Publication of this article was funded by Natural Science and Engineering Research Council of Canada (NSERC).

This article has been published as part of *BMC Systems Biology *Volume 8 Supplement 3, 2014: IEEE International Conference on Bioinformatics and Biomedicine (BIBM 2013): Systems Biology Approaches to Biomedicine. The full contents of the supplement are available online at http://www.biomedcentral.com/bmcsystbiol/supplements/8/S3.
